# Power and Weakness of Repetition – Evaluating the Phylogenetic Signal From Repeatomes in the Family *Rosaceae* With Two Case Studies From Genera Prone to Polyploidy and Hybridization (*Rosa* and *Fragaria*)

**DOI:** 10.3389/fpls.2021.738119

**Published:** 2021-12-07

**Authors:** Veit Herklotz, Aleš Kovařík, Volker Wissemann, Jana Lunerová, Radka Vozárová, Sebastian Buschmann, Klaus Olbricht, Marco Groth, Christiane M. Ritz

**Affiliations:** ^1^Department of Botany, Senckenberg Museum of Natural History Görlitz, Görlitz, Germany; ^2^Department of Molecular Epigenetics, Institute of Biophysics, Academy of Sciences of the Czech Republic, Brno, Czechia; ^3^Institute of Botany, Systematic Botany Group, Justus-Liebig-University, Gießen, Germany; ^4^Department of Experimental Biology, Faculty of Science, Masaryk University, Brno, Czechia; ^5^Institute of Botany, Technische Universität Dresden, Dresden, Germany; ^6^Hansabred GmbH & Co. KG, Dresden, Germany; ^7^CF DNA Sequencing, Leibniz Institute on Aging – Fritz Lipmann Institute, Jena, Germany; ^8^Chair of Biodiversity of Higher Plants, Technische Universität Dresden, International Institute (IHI) Zittau, Zittau, Germany

**Keywords:** high-throughput sequencing, graph-based clustering, repeatome, repetitive DNA, phylogenetics, *Rosaceae*, *Fragaria*, *Caninae*

## Abstract

Plant genomes consist, to a considerable extent, of non-coding repetitive DNA. Several studies showed that phylogenetic signals can be extracted from such repeatome data by using among-species dissimilarities from the RepeatExplorer2 pipeline as distance measures. Here, we advanced this approach by adjusting the read input for comparative clustering indirectly proportional to genome size and by summarizing all clusters into a main distance matrix subjected to Neighbor Joining algorithms and Principal Coordinate Analyses. Thus, our multivariate statistical method works as a “repeatomic fingerprint,” and we proved its power and limitations by exemplarily applying it to the family *Rosaceae* at intrafamilial and, in the genera *Fragaria* and *Rosa*, at the intrageneric level. Since both taxa are prone to hybridization events, we wanted to show whether repeatome data are suitable to unravel the origin of natural and synthetic hybrids. In addition, we compared the results based on complete repeatomes with those from ribosomal DNA clusters only, because they represent one of the most widely used barcoding markers. Our results demonstrated that repeatome data contained a clear phylogenetic signal supporting the current subfamilial classification within *Rosaceae*. Accordingly, the well-accepted major evolutionary lineages within *Fragaria* were distinguished, and hybrids showed intermediate positions between parental species in data sets retrieved from both complete repeatomes and rDNA clusters. Within the taxonomically more complicated and particularly frequently hybridizing genus *Rosa*, we detected rather weak phylogenetic signals but surprisingly found a geographic pattern at a population scale. In sum, our method revealed promising results at larger taxonomic scales as well as within taxa with manageable levels of reticulation, but success remained rather taxon specific. Since repeatomes can be technically easy and comparably inexpensively retrieved even from samples of rather poor DNA quality, our phylogenomic method serves as a valuable alternative when high-quality genomes are unavailable, for example, in the case of old museum specimens.

## Introduction

In most eukaryotic genomes, especially in higher plants, the majority of nuclear DNA consists of repetitive elements, which, in total, are referred to as repeatome (Biscotti et al., 2015). However, the expected correlation between the amount of DNA per nucleus (*C*-value) and the complexity of an organism is often violated among closely related species (Choi et al., 2020). Repetitive elements show a huge variability across taxa in terms of structure, quantity, and chromosomal positions (Brookfield, 2005; Dodsworth et al., 2015). Tandemly repeated satellite DNAs (satDNA), such as ribosomal RNA genes (rDNA) or centromeric satellites, occur in long arrays of similar motifs located on a limited number of loci in relatively specific chromosome domains. In contrast, transposable elements (TEs) are highly variable, mostly dispersed throughout the genome (Biscotti et al., 2015; Bourque et al., 2018), and transferred and amplified by DNA (Class II TE) or *via* an intermediate RNA (Class I TE). In addition, within these TE classes, an amazing variety of types can be classified, and the abundance of certain types differs highly between taxa (Leitch and Leitch, 2008; Biscotti et al., 2015; Wendel et al., 2016). Furthermore, genomes can be seen as “ecosystems” occupied by numerous TE populations aiming to expand and reproduce by dynamic interactions with each other and with other cell components (Venner et al., 2009). In addition, there is significant evidence for the hypothesis that horizontal TE transfer is widespread (Gilbert and Feschotte, 2018; Wallau et al., 2018).

A considerable part of the repeatome is accounted for ribosomal DNAs. In particular, the 45S rDNA and the 5SrDNA are organized in large distinct loci on several chromosomes, which can be relatively easy visualized by Fluorescent *in situ* Hybridization (Schwarzacher and Heslop-Harrison, 2000). This has been widely applied to elucidate chromosomal evolution, especially tracking polyploidy and hybridization in many plant groups, including *Rosaceae* (Kovařík et al., 2004; Liu and Davis, 2011; Herklotz et al., 2018). Ribosomal DNA loci are composed of hundreds to thousands of tandemly repeated sequence units, which are homogenized by several mechanisms such as gene conversion and unequal crossovers summarized under the term concerted evolution (Wissemann, 2003; Eickbush and Eickbush, 2007). Their multi-copy nature, their ubiquitous presence across genomes, and their highly conserved genes within the arrays have made particularly the non-coding parts, among others, the internal transcribed spacer sequences (ITS), to standard barcoding markers in plants over decades. However, due to the presence of pseudogenes and paralogous sequences, ITS markers turned out to be phylogenetically misleading in numerous cases (Álvarez and Wendel, 2003; Poczai and Hyvönen, 2010). Delayed rDNA homogenization between subgenome has been proved as a valuable tool for tracking parental lineages in hybrids (e.g., Wissemann, 1999, 2002; Devos et al., 2005; Mlinarec et al., 2012).

High throughput sequencing approaches with low coverage such as genome skimming (0.1–5× coverage) represent straightforward and cost-effective methods to analyze repeatomes. The RepeatExplorer2 (RE) pipeline characterizes *de novo* genomic repeats by graph-based clustering (Novák et al., 2010, 2013, 2020) and allows the simultaneous analysis of multiple samples (e.g., species, individuals). Dodsworth et al. (2015) used such comparative RE clustering to track phylogenetic signals from repeatomes by counting the number of reads per species in each cluster. Vitales et al. (2020) further developed this method by calculating pairwise genetic distances from each cluster and subsequently computing neighbor-joining trees per cluster, which were then summarized into a consensus tree. Based on these studies, we now suggest new adjustments of this approach. In contrast to Dodsworth et al. (2015) and Vitales et al. (2020), who used RE input reads in direct proportion to the genome size in order to reflect the proportion of repeat abundance per genomes, we propose here to adjust the read input amount in indirect proportion to the genome size to overcome the biased self-interconnection in graph-based clustering for species with large genomes and high repeat abundance. In addition, we summarize all dissimilarities for each cluster in a main distance matrix, which can then be used for various multivariate statistical approaches.

**FIGURE 1:**
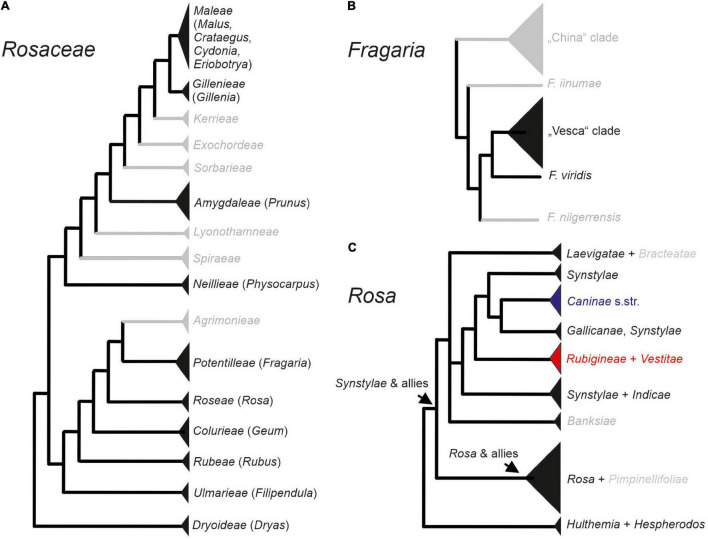
Phylogenetic relationships at higher taxonomic levels within *Rosaceae* **(A)**, *Fragaria* **(B)**, and *Rosa* **(C)**. Within the genus *Rosa*, sect. *Caninae* represented by subsections *Caninae* (blue), *Rubigineae*, and *Vestitae* (red) form a separate data set for the analysis. Taxa depicted in gray were not represented in our study.

Being a medium-sized family of 92 genera and 2,805 species (Stevens, 2001) mainly distributed in the temperate regions of the Northern Hemisphere, *Rosaceae* are one of the most remarkable examples for polyploid evolution (Dickinson, 2018). During the last years, phylogenetic relationships at higher taxonomic levels within *Rosaceae* have been rather consolidated because data from plastomes and nuclear low copy genes subdivide the family into three subfamilies: *Dryadoideae*, *Rosoideae*, and *Amygdaloideae* (Xiang et al., 2016; Zhang et al., 2017). The latter contains not only the apple and plum-fruited tribes (*Maleae, Amygdaleae*) but also dry-fruited species (e.g., *Spiraeae*; see Figure 1A). However, major challenges do still exist for relationships within the mainly polyploid tribe *Maleae* (Lo and Donoghue, 2012; Sun et al., 2018).

In the economically highly important genera *Rosa* (roses) and *Fragaria* (strawberries) of subfamily *Rosoideae*, speciation has been mainly driven by allopolyploidy. The herbaceous genus *Fragaria* contains 23 species, and most polyploid members evolved gender dimorphism, ranging from gynodioecy to dioecy, whereas diploids are mostly hermaphroditic (Liston et al., 2014). The genus is divided in two major lineages: the *F. vesca* clade and the China clade, in addition to some species with unresolved position, among them the Eurasian species *F. viridis* (Figure 1B; Rousseau-Gueutin et al., 2009). The Eurasian hexaploid (2*n* = 6*x* = 42), dioecious *F. moschata*, is assumed to be of allopolyploid origin. However, data on parental lineages of this species have been conflicting: Nuclear and plastid sequences supported a hybridization scenario between *F. viridis* (maternal parent) and *F. vesca* (Lin and Davis, 2000; Rousseau-Gueutin et al., 2009). In contrast, plastome data support the maternal origin from the *F. vesca* clade and did not provide evidence for the involvement of *F. viridis* (Njuguna et al., 2013). The first hypothesis is additionally supported by the presence of the rather abundant spontaneous hybrids between *F. vesca* and *F. viridis*, namely *F.* ×*bifera* (Staudt et al., 2003).

The woody plant genus *Rosa* comprises ca. 150 species and is, similar to *Fragaria*, separated into two major clades: *Synstylae* and allies and *Rosa* and allies, as well as some species-poor or monotypic subgenera at the basal position: subg. *Hulthemia*: *R. persica* and subg. *Hesperhodos*: *R. minutifolia* (Figure 1C; Fougère-Danezan et al., 2015; Debray et al., 2021). Nested within the *Synstylae* and allies clade that appear to be members of sect. *Caninae*, the dogroses, contain approximately 30 species. This enigmatic group of plants consists exclusively of polyploids, most of them with an odd chromosome number being pentaploid (2*n* = 5*x* = 35), and they presumably evolved by multiple hybridization events (Ritz et al., 2005). Despite the fact that recent data point to a polyphyletic origin of the group with subsect. *Caninae* separated from subsect. *Rubigineae* and *Vestitae* (Herklotz et al., 2018; Lunerová et al., 2020; Vozárová et al., 2021), all members are characterized by an asymmetric meiosis system, namely the *Canina* meiosis (Blackburn and Harrison, 1921; Täckholm, 1922). During the course of this meiosis, only two subgenome form bivalents, whereas the other chromosome sets remain unpaired. As a result of sex-specific meiotic movements, pollen grains contain seven chromosomes (one set) and egg cells 28 chromosomes (four sets) so that their fusion restores the odd somatic number (Täckholm, 1922). This leads to strong matroclinal inheritance where 80% of the genome is of maternal origin, and only 20% comes from the paternal parent.

During this study, we want to use repeatomes from *Rosaceae* to explore to which extent these data can be used to track phylogenetic signals at various taxonomic levels, taking polyploidy and hybridization into account. In contrast to the previous methods, we conducted our phylogenetic reconstructions and multivariate statistical approaches based on the sum of all cluster dissimilarities using them as a “repeatomic fingerprint” (see details below). To test our adapted approach for its power in detecting phylogenetic signals at different taxonomic levels, we used entire repeatome and rDNA data from the family *Rosaceae* as an example. More specifically, we addressed the following questions: (1) Does the here proposed modified method of repeatome analysis provide useful information on phylogenetic relationships at a wide taxonomic level (within major clades of *Rosaceae*) and at lower taxonomic levels (between species of *Fragaria* and *Rosa*, respectively). (2) Do repeatome data allow insights into hybridization events, namely into the hybridogenic origin of *F. moschata* and of *Rosa* sect. *Caninae*, respectively? (3) Are results based on complete repeatomes comparable to those obtained from ribosomal DNAs as a typical example for a dominant and widely used part of repeatomes?

## Materials and Methods

### Plant Material and Short Read Data

For tracing phylogenetic relationships within *Rosaceae*, we sampled short reads of 24 species, reflecting the subfamilies and major tribes of *Rosaceae* from the Sequence Read Archive (SRA, data set “*Rosaceae*,” Supplementary Table 1). Criteria for selection of suitable Illumina reads were a genomic DNA-based sequencing approach and random library selection.

In order to study the repeatomes within *Fragaria*, we sampled fresh leaf material from 12 plants from the “*Professor Staudt Collection*” (Olbricht et al., 2014) hosted by Hansabred GmbH & Co. KG, Dresden, Germany (Dataset “*Fragaria*,” Supplementary Table 1). Sampling included representatives of the *F. vesca* clade, namely one individual each of *F. bucharica*, *F. mandshurica*, and *F. orientalis*, three individuals each of *F. vesca* and *F. viridis* and the proposed hybridogenic species *F. moschata*. Additionally, we sampled triploid and diploid individuals each of *F.*×*bifera*, constituting a naturally occurring hybrid between *F. vesca* × *F. viridis* (Staudt et al., 2003; Tushabe, 2019). Accession numbers and sampling details are listed in Supplementary Table 1.

For analyzing phylogenetic relationships within *Rosa*, we sampled 14 members of the genus from ENA (data set “*Rosa*,” Supplementary Table 1). To follow the hybridogenic origin of sect. *Caninae*, we sampled three members of subsect. *Caninae*, two of subsect. *Rubigineae* and one of subsect. *Vestitae*. In addition, we newly generated repeatome data from synthetic hybrids between subsections *Rubigineae* and *Caninae* (data set “*Caninae*,” Supplementary Table 1) obtained from the Botanical Garden Gießen, Germany (Wissemann and Hellwig, 1997).

### DNA Extraction, High-Throughput Sequencing, and Data-Base Accessions

Genomic DNA from *Fragaria* samples and *Caninae* synthetic hybrids (Supplementary Table 1) were isolated from silica-gel dried leaflets using the ATMAB protocol (Dumolin et al., 1995). Subsequently, high molecular weight DNA was purified with a Mag-Bind^®^ Plant DNA DS kit (Omega Bio-tek, Norcross, United States) and quantified with Qubit4 Fluorometer (Life Technologies). Library preparation using NEBNext^®^ DNA Library Prep Kit with an insert size of 350 bp and Illumina sequencing in a 150 bp paired-end mode were done by Novogene Europe (Cambridge, United Kingdom). *Fragaria* samples were sequenced at the Leibniz Institute on Aging – Fritz Lipmann Institute (Jena, Germany). Sequencing of DNA samples was performed using Illumina’s next-generation sequencing methodology (Bentley et al., 2008). In detail, genomic DNA was quantified using the Quant-iTPicoGreen dsDNA Assay Kit (Invitrogen). Prior to library preparation, genomic DNA was fragmented to around 450 bp using Covaris M220. Libraries were prepared from 100 ng of input material using NEBNext Ultra II Directional RNA Library Preparation Kit, including size-selection (400–500 bp), following the manufacturer’s instructions (New England Biolabs Inc., MA, US). Quantification and quality check of libraries were done using Agilent 2100 Bioanalyzer Instrument and a DNA 7500 assay (Agilent Technologies, Santa Clara, CA, United States). Libraries were pooled and sequenced on a NextSeq 500 mid-output 300 cycle v2.5 run. System run in a 151-cycle/paired-end workflow mode. Sequence information was converted to FASTQ format using bcl2fastq v2.20.0.422.

### RepeatExplorer2 Comparative Clustering

#### Methodological Background

The RepeatExplorer2 (RE) pipeline classifies genomic repeats by quantifying sequence similarities between short reads (100–300 bp). Because of random genomic sampling, these short reads represent highly abundant repeat sequences (Novák et al., 2010, 2013, 2020). The first step is an all-to-all pairwise BLAST comparison (Altschul et al., 1990), capturing all read pairs with sequence overlaps that surpass a specified threshold (90% similarity over ≥55% of the read length). Based on this, a large virtual graph is computed in which nodes correspond to sequence reads, while overlapping reads are linked by edges (Novák et al., 2010). The underlying network construction in RE is an intermediate step utilizing the iGraph package (Yu et al., 2009). Separating communities of similar reads into clusters is done by a graph-based clustering algorithm (Novák et al., 2010) using the Louvain modularity optimization method for community detection (Blondel et al., 2008).

This method can also be extended to phylogenetic studies across multiple taxa (Dodsworth et al., 2015; Vitales et al., 2020). Using RE in a comparative mode, i.e., between taxa, the same repeat family can be found in different taxa. Thus, related reads from different taxa can be placed into the same cluster, and clusters containing reads from only one taxon represent taxon-specific repeats. For comparative RE clustering between species with large differences in genome size and repeat abundance, the number of analyzed reads should be adjusted. Vitales et al. (2020) proposed a distance-based method for extracting phylogenetic signals from RE data where the RE pipeline generates index.html files for each cluster. These files include a first matrix with counts of significant BLAST matches between the reads of the different species, which is reflected by the number of actual observed edges in the cluster graph. In addition, Vitales et al. (2020) used a second matrix in the index.html files containing the proportions between the observed and expected number of edges for each species pair (Novák, 2019, lines 1245–1268). The expected number of edges is calculated in RE by matrix multiplication of the proportion of edges (number of edges per species pair/total number of edges in the cluster) to each other (Novák, 2019, line 1253). This parameter normalizes for unequal representation of reads from different species in that cluster. Thus, this pairwise matrix of observed/expected numbers of edges takes the different repeat abundances between species per cluster into account and can be considered as a mean of true sequence similarities between repeats of different species origins. Vitales et al. (2020) treated the observed/expected numbers of edges as a pairwise similarity matrix for each cluster and transformed them into distance matrices by simple inversion. Then, neighbor-joining algorithm was applied for each of the top 100 clusters, and, subsequently, a consensus tree was calculated. In contrast to this, we summarized all matrices of the inverted observed/expected number of edges (displaying the majority of repeats) into one main “obs”-distances matrix. Additionally, we also inverted and summarized the first matrices in the index.htmls containing the actual number of edges in order to analyze the unweighted distribution of dissimilarities. We call this the “edges”-distance matrix.

**FIGURE 2:**
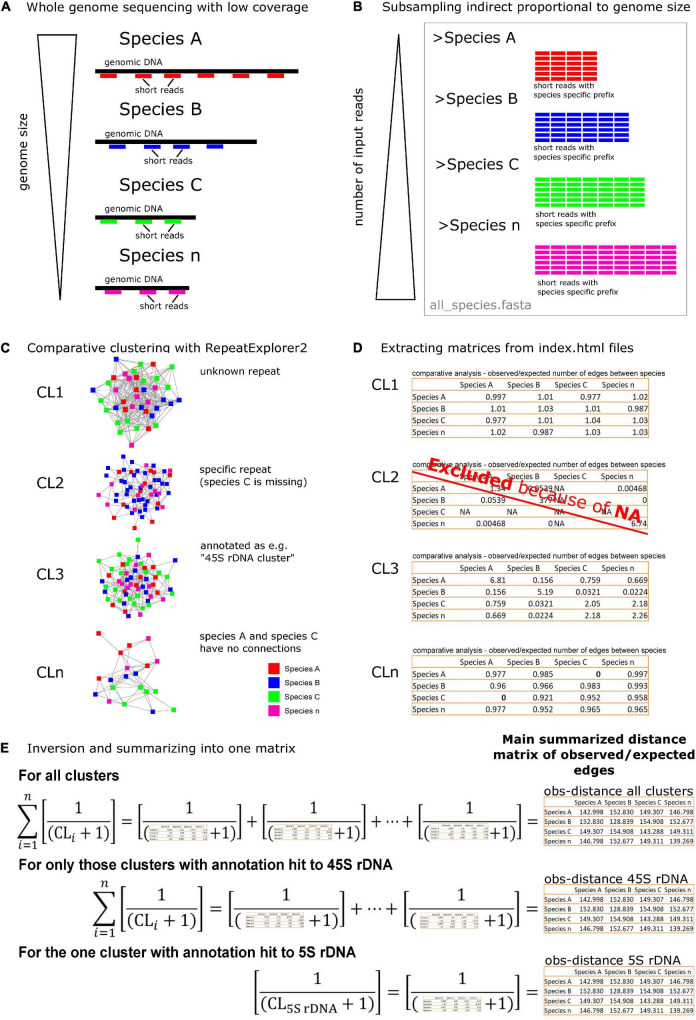
A schematic overview of the method used to analyze phylogenetic signals in repeatome data. **(A)** The comparative study was based on Illumina short read sequences from different species with unequal genome sizes. **(B)** Reads from different species were concatenated with indirect proportional number to their genome sizes, creating one FASTA file **(C)**. A comparative clustering with the RepeatExplorer2 (RE) pipeline was conducted on a mixed data set. The resulting graphs reflected shared repeat sequences with nodes representing (species-specific) reads and in-between edges showing their similarity. **(D)** A bash script (Supplementary File 1) was used to extract pairwise similarity matrices of each cluster index.html out of the RE archives. The matrix values represented the ratio between the number of observed and expected edges in a cluster graph. Matrices containing missing reads in a species for a specific cluster (NAs) were excluded. **(E)** Finally, matrices were inverted, and 1 was added to each matrix value to avoid zero values due to missing connections between species within a shared repeat (Supplementary File 2). Sub-data sets were built from only those clusters with 45S rDNA hits or the 5S rDNA clusters. The resulting summarized matrices served as distance and variable tables for further statistics. The same procedure was done for the number of edges matrices (Supplementary Figures 1, 2).

Both previous studies (Dodsworth et al., 2015; Vitales et al., 2020) used RE input reads in direct proportion to the genome size in order to reflect repeat abundance per genome. We were interested in dissimilarities between species reflected by the number of edges, not in the repeat abundance reflected by the number of nodes. Therefore, we used the read input amounts in indirect proportion to the genome size (Figures 2A,B and Supplementary Table 1).

#### Data Pre-treatment and RepeatExplorer2 Settings

Four separate comparative clustering analyses (Supplementary Table 1) were conducted (“*Rosaceae”*: 4,738,391 reads, “*Fragaria”*: 2,251,549 reads, “*Rosa”*: 1,298,057 reads, *“Rosa* sect. *Caninae”*: 2,600,000 reads). All members of *Rosa* sect. *Caninae* investigated here were pentaploid (2*n* = 5*x* = 35) and of same genome size (Ritz and Wissemann, 2011); thus, we used an equal number of input reads for each individual. Quality trimming and adapter removal were done for all reads using Geneious^®^ 10.0.9,^1^ and reads were trimmed to the first 100 nucleotides. Finally, read names were tagged with a species-specific four-character long prefix and concatenated into one FASTA file per data set (Figure 2B). Advanced settings for the RE pipeline were as follows: single-end reads (because paired-end reads were not available for all species), perform comparative analysis, group code length of 4, perform automatic filtering of abundant satellite repeats, and a long queue (max run time, 2 weeks; 65 Gb RAM).

### Data Extraction From RepeatExplorer2 Archives

The compressed RE archives were downloaded from the Galaxy server,^2^ and the unique folder and output structure of RE archives enabled us to extract cluster-specific information.

The comparative RE pipeline summarizes the results for each cluster in an index.html file (./*Archive*/seqclust/clustering/clusters/dir_CL[n]/index.html). This file contains, among other information, a pairwise matrix with the actual observed number of edges (later referred to as “edges”) between species and a pairwise matrix with ratios between the observed and the expected number of edges (later referred to as “obs”). We applied the bash command *html2txt* (Unkrig, 2004; Groffen, 2021) to all cluster’s index.html files for converting them into text files. Subsequently, both matrices were copied from the text file into separate files stored in the subfolders “edges” and “obs”, respectively. For species-specific clusters containing reads of only one species, RE could not build a matrix connecting species by edges. Those matrix files were filtered out by 0-kb size criteria. If a species was missing in a certain cluster (no reads of this species in a cluster), RE marked this as “NA” (not applicable, dividing by zero) in the obs-matrices. Although we lost specific information, all incomplete obs-matrices containing “NA” were removed from further analyses (according to Vitales et al., 2020; Figure 2D) because we aimed for a comparative analysis between common repeats. Additionally, clusters annotated with “contamination” because of RE-detected adapter sequences were filtered out. All these operational steps were automatically done by executing a bash script (Supplementary File 1) from the superior directory of the archive directory.

### Multivariate Statistics

Statistical analyses were run under the R environment (R Core Team, 2020; RStudio Team, 2021). For each of the four RE analyses, all edges- and obs-matrices were loaded separately from text files as a list using *lapply*. To avoid zero values caused by the lack of edges between species (although their reads were in the cluster), each value was added by 1 (Figures 2C,E and Supplementary File 2). According to Vitales et al. (2020), all similarity matrices were transformed into dissimilarities by inversion. Subsequently, we summarized all cluster obs-matrices to generate a master obs-matrix using the *Reduce* function (Supplementary File 2) for each of the four RE analyses. In addition, we did this procedure (Figure 2) also for the edges matrices. These summarized main obs- and edges- matrices were treated directly as distance according to Vitales et al. (2020), square-rooted, and used for Principal Coordinate Analyses (PCoA). On the other hand, the main obs-matrices were treated as data table with variables and used for neighbor-joining algorithms. Ordination graphs were drawn with *ggplot2* (Wickham, 2016). Additionally, clusters with hits >1% to 45S rDNA and 5S rDNA were analyzed separately. For the “*Rosaceae*” data set, we omitted the rDNA analysis because of too divergent clusters and a high number of NAs in obs-matrices. To present the general phylogenetic signal of these main matrices in a hierarchical manner, we applied neighbor-joining (NJ) trees with 1,000 bootstrap replicates to the “*Rosaceae*” data set based on Gower distances with MEGA X, *vegan* and *ape* packages (Kumar et al., 2018; Paradis and Schliep, 2019; Oksanen et al., 2020). Note that the Gower distance (Gower, 1971) based on numerical data like in our case is identical to range normalized Manhatten-distance. Since the displayed ordinations visualize only the first two axes, they represent only a part of explaining variance. To account for the full amount of variation in the data sets, we additionally calculated neighbor nets based on Gower distances for the data sets “*Fragaria*,” “*Rosa*,” and “*Caninae*.” Neighbor nets allow for the detection of hybridization patterns and were drawn with *phangorn* 2.6.2 (Schliep et al., 2017).

## Results

### Rosaceae

**FIGURE 3:**
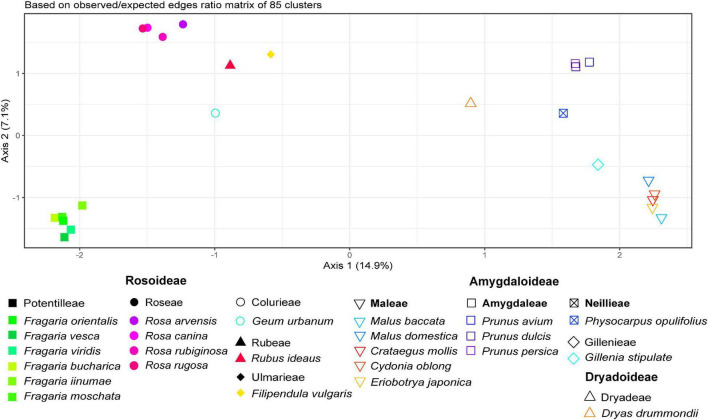
Principal Coordinate Analysis of *Rosaceae* species based on 85 remaining inverted and summarized observed/expected matrices from the comparative RepeatExplorer2 output after removal of matrices containing NAs. Axis percentages explain the variance on the first two dimensions. Colors represent species and symbols taxonomic affiliations.

Samples from 24 *Rosaceae* species across three subfamilies and 10 tribes were comparatively analyzed with RE, resulting in 352 clusters. In summary, 33% of the 4.7 million analyzed reads were classified as repetitive elements. For further analysis, Cluster 1 was removed due to suspected prokaryotic sequence contamination in reads of *Physocarpus opulifolius*. Additionally, we removed 28 species-specific clusters, containing only reads from one species and one cluster with an (adaptor) “contamination” hit. The majority of clusters missed at least one species (resulting in NAs) and thus had to be omitted, which resulted in a summarized obs-distance matrix based on 85 clusters (Figure 3). In addition, the PCoA in Supplementary Figure 1 represents dissimilarities across the remaining 322 clusters based on main summarized edges distance.

**FIGURE 4:**
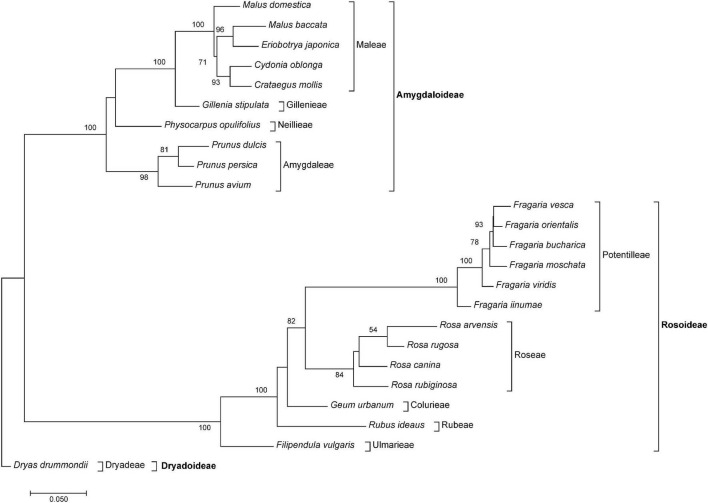
The neighbor-joining tree of *Rosaceae* species based on Gower distance calculated from the sum of 85 obs-distances (inverted observed/expected numbers of edges matrices remaining after removal of matrices containing NAs) obtained from the comparative RepeatExplorer2 output. Bootstrap percentages >50% are given above branches. The tree was rooted with *Dryas drummondii*. Subfamilies and tribes are indicated after brackets.

The PCoA in Figure 3 reflects relationships among major clades within *Rosaceae*, and species belonging to the same genus were close to each other. Members from the tribe *Amygdaloideae* were clustered on the right and those of *Rosoideae* on the left side of the plot. *Dryas* (tribe *Dryadoideae*) was located in between but closer to the *Amygdaloideae*. Within the *Amygdaloideae*, members of the tribe *Maleae* (*Crataegus, Cydonia, Eriobotrya*, and *Malus*) were arranged in close proximity. The PCoA based on the summarized edges distances showed a similar grouping of taxa (Supplementary Figure 1). Accordingly, the neighbor-joining trees based on Gower distances from both summarized edges- and obs-distances (Figure 4 and Supplementary Figure 2) showed known phylogenetic relationships within *Rosaceae* by placing subfamilies into two separate clades, albeit Bootstrap support was rather low for some branches within subfamilies. Within sub fam. *Amygdaloideae*, the tribe *Amygdaleae* and *Physocarpus opulifolius* (*Neillieae*—but only 44% Bootstrap support) were sisters to a clade formed by *Gillenia stipulata* (*Gillenieae*) and members of the *Maleae*. Within subfam. *Rosoideae*, the branching order from the base was first *Filipendula* (*Ulmarieae*), followed by *Rubus* (*Rubeae*), *Geum* (*Colurieae), Rosa (Roseae*), and *Fragaria (Potentilleae*). The neighbor-joining tree based on the sum of 322 edges distances (Supplementary Figure 2) showed a congruent topology, but *Physocarpus* (*Neillieae*) was in the basal position to all other members of *Amygdaloideae*.

### *Fragaria (Potentilleae*)

**FIGURE 5:**
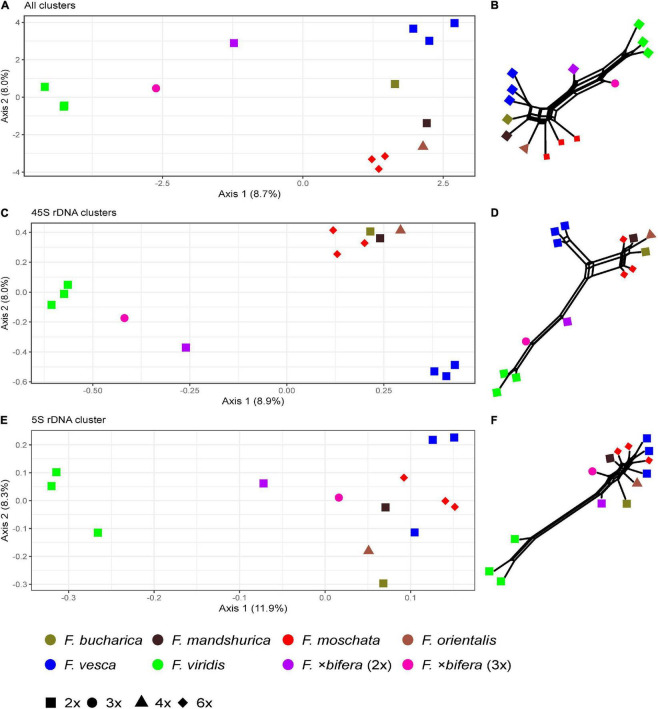
Principal Coordinate Analyses of *Fragaria* species based on the inverted and summarized observed/expected matrices from comparative RepeatExplorer2 output and neighbor nets based on Gower distances calculated from those comparative RepeatExplorer2 outputs. Axis percentage explains the variance on the first two dimensions. **(A)** PCoA and **(B)** the neighbor net across all 352 clusters, **(C,D)** across seven clusters containing 45S rDNA, and **(E,F)** Cluster 188 containing 5S rDNA hits.

**FIGURE 6:**
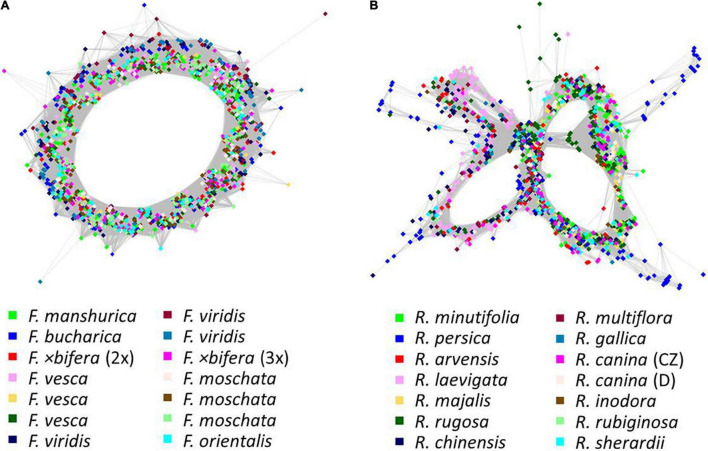
Graph shapes of 5S rDNA clusters from RepeatExplorer2 comparative clustering. Colors indicate **(A)** *Fragaria* and **(B)** *Rosa* species. Nodes represent reads, and their similarities are expressed as edges. The general graph shape depends on the repeat motif and its variability.

The comparative RE analysis of 14 individuals from seven *Fragaria* species revealed 352 major clusters. In total, 33% of the 2.7 million analyzed reads were assigned to repetitive elements. All clusters were retained because no NAs were found in obs-matrices and no (adaptor-) “contamination” was detected. The PCoA based on the main summarized obs-distance matrix across all clusters (Figure 5A) separated the three individuals of *F. viridis* from all other samples along the first axis. Along the second axis, *F. vesca* was separated from *F. moschata*, and the Asian species *F. orientalis*, *F. bucharica*, and *F. mandshurica* were located between the *F. moschata* and *F. vesca* (Figure 5A). Both individuals of *F.*×*bifera* appeared in intermediate positions between *F. vesca* and *F. viridis*. The triploid sample of *F.*×*bifera* was closer to *F. viridis* along Axis 1, whereas the diploid *F.*×*bifera* was located between its parental species. The PCoAs of seven clusters with hits to 45S rDNA and one cluster of 5S rDNA, respectively, showed a similar grouping of species (Figures 5C,E). In general, *F. moschata* was closer to Asian *Fragaria* species than to *F. viridis* and *F. vesca.* In the 5S rDNA analysis, one individual of *F. vesca* appeared separate from the other sample *F. vesca* but was closer to Asian species (Figure 5E). The explaining variance for the first two axes of the PCoA was not more than 20%, but the overall pattern was similar to that obtained by the respective neighbor nets (Figures 5B,D,F). The RE graph of the cluster with 5S rDNA hits showed a distinct ring-like shape (Figure 6A). The PCoA based on the main summarized edges distance did not show any species-specific signals but was characterized by single outliers. The majority of samples clustered in the center of the ordination without any detectable separation, while three to four individuals, not always belonging to the same species, were strongly detached (data not shown).

### *Rosa (Roseae*)

**FIGURE 7:**
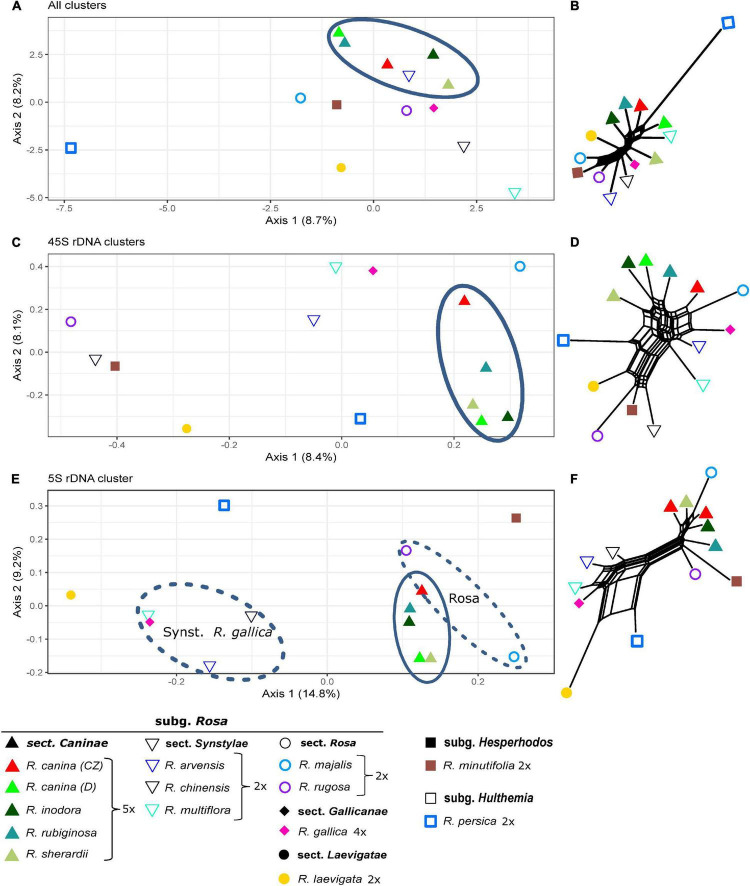
Principal Coordinate Analyses of *Rosa* species based on inverted and summarized observed/expected matrices from comparative RepeatExplorer2 output and neighbor nets based on Gower distances calculated from those comparative RepeatExplorer2 outputs. Axis percentages explain the variance on the first two dimensions. **(A)** PCoA and **(B)** the neighbor net across all 297 clusters, **(C,D)** only those four with 45S rDNA hits, and **(E,F)** Cluster 107 with 5S rDNA hits. Oval – *Rosa* section *Caninae*. The oval dashed line indicating *Synstylae*, *R. gallica*, and the sect. *Rosa* phylogenetic signal in the 5S rDNA cluster.

The comparative RE analysis of 14 rose species revealed 310 clusters. In total, 40% of the 1.5 million analyzed reads were assigned to repetitive elements. Five clusters were removed because they were annotated with (adaptor-) “contamination,” and, additionally, eight obs-matrices were omitted because of the presence of NAs. Four of them were either absent or nearly species-specific, i.e., containing almost only reads from *R. persica* and very few reads from other species; the remaining four were either specific for some species of the *Synstylae* and allies clade (*R. multiflora, R. arvensis, R. gallica*, and *R. rubiginosa*) or lacked these species. Based on the main summarized obs-distance matrix, we calculated a PCoA and a neighbor net representing dissimilarities across all remaining 297 clusters (Figures 7A,B). *Rosa persica* (subg. *Hulthemia*) was clearly separated from the remaining samples, but *R. minutifolia* (subg. *Hesperhodos*), *R. rugosa*, and *R. majalis* (subg. *Rosa* sect. *Rosa*) were intermingled with species from the *Synstylae* and allies clade (sect. *Caninae*, *Gallicanae*, *Laevigatae*, and *Synstylae*). However, the European species of the *Synstylae* and allies clade (2x *R. arvensis*, 4x *R. gallica* as well as all members of the pentaploid dogroses; the latter highlighted by an oval) were closely clustered in the PCoA. Similarly, the PCoA (Figure 7C), and the neighbor net (Figure 7D) based on data from the four clusters with hits to 45S rDNA showed no clear pattern concerning major lineages, but dogroses were also grouped somewhat closer. The PCoA (Figure 7E) and the neighbor net (Figure 7F) based on one 5S rDNA-related cluster showed members of the section *Caninae* in close proximity to each other on the right side together with members of sect. *Rosa* (*R. majalis* and *R. rugosa*), whereas the remaining members of the *Synstylae* and allies clade were located on the left side in the PCoA (Figure 7E). The RE graph of the cluster with 5S rDNA hits showed a four-loop structure with some species-specific loops, indicating a more heterogeneous 5S rDNA non-transcribed spacer (Figure 6B). The analogous PCoA based on the sum of edges distances lacked any separation between taxa except for single outliers. The majority of samples appeared densely arranged in the center of the plot, while some samples were separated along the axes without any apparent pattern in the respective subsets (45S rDNA, 5S rDNA; data not shown).

### *Rosa* Sect. *Caninae*

**FIGURE 8:**
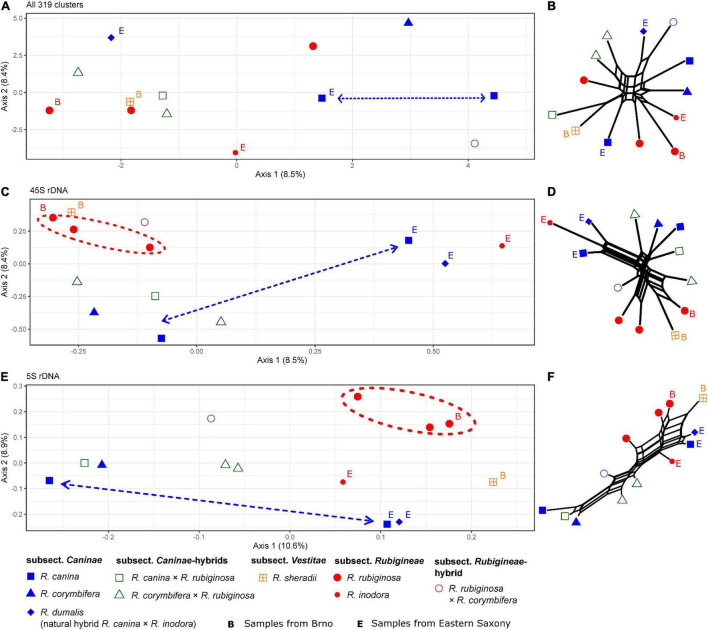
Principal Coordinate Analyses of *Rosa* sect. *Caninae*, including natural and synthetic hybrids based on inverted and summarized observed/expected matrices from the comparative RepeatExplorer2 output and neighbor nets based on Gower distances calculated from those comparative RepeatExplorer2 outputs. Axis percentages explain the variance on the first two dimensions. **(A)** PCoA and **(B)** the neighbor net over all 319 clusters. **(C,D)** Only those five clusters with 45S rDNA hits and **(E,F)** cluster 108 with 5S rDNA hits. Unlabeled parental individuals and artificial hybrids were originally from Göttingen (Lower Saxony, Germany). The individuals from Eastern Saxony (Germany), including the natural hybrid *R. dumalis*, were labeled with **(E)** and roses from Brno (Czechia) with **(B)**. Note the clustering of *R. rubiginosa* (red) but not *R. canina* (blue) in all rDNA and graphs **(C,D,E,F)**. Large distances between *R. canina* indicated by dashed arrows and close *R. rubiginosa* by dashed circles.

The comparative RE analysis of 13 individuals from *Rosa* sect. *Caninae* resulted in 319 major clusters. In total, 43% of the 2.6 million analyzed reads were assigned to repetitive elements. No clusters were removed because of “NAs” in obs-matrices or annotations with (adaptor-) “contamination.” The PCoA and the neighbor net did not reveal a clear phylogenetic pattern (Figures 8A,B) because members of the subsections *Caninae, Rubigineae*, and *Vestitae* were not separated from each other, and we observed quite large distances between samples of the same species. Moreover, matroclinal synthetic hybrids were not clustered close to their mothers. Contrarily, the graphs based on 45S rDNA separated the samples according to their geographical origin along the first axis in the PCoA (Figure 8C) and in the neighbor net (Figure 8D). The samples *R. canina* and *R. inodora* from Eastern Saxony (labeled with “E”) were clustered together in the right part of the diagram (Figure 8C), with their natural hybrid from the same locality, *R. dumalis* (*R. canina* × *R. inodora*) occupying an intermediate position between its parental species. On the left part of the diagram, the remaining samples from Lower Saxony and *R. sherardii* (subsect. *Vestitae*) were clustered. Members of subsect. *Rubigineae* (except *R. inodora*) were in proximity to the hybrid *R. rubiginosa* × *R. canina* (Figures 8C,D). Species of subsect. *Caninae* (*R. canina* and *R. corymbifera*) were clustered together with their respective synthetic matroclinal hybrids. A similar pattern was found in the graphs based on 5S rDNA sequences (Figures 8E,F). Samples from Eastern Saxony were arranged in the lower right corner of the PCoA with *R. dumalis* being close to its maternal species *R. canina*. Samples from Lower Saxony were spread along the first axis: The parental species from subsects. *Caninae* and *Rubigineae* were widely separated, and synthetic hybrids were located in between. In general, we found closer clustering among samples of the parental species *R. rubiginosa* compared to those of *R. canina* in both rDNA analyses (Figures 8C,D). The neighbor nets (Figures 8B,D,F) based on Gower distances calculated from the obs-matrices were in general concordant to the patterns detected on the first two axes of PCoAs.

## Discussion

Repeatomes among individuals of the same species can be highly diverse (Biscotti et al., 2015; Bourque et al., 2018) and may even serve as individual-specific fingerprints (Negm et al., 2020). Still, repeatomes may contain phylogenetic information at various levels ranging from populations to higher taxonomic ranks (Dodsworth et al., 2015; Bolsheva et al., 2019; Negm et al., 2020; Vitales et al., 2020; Dogan et al., 2021). In order to investigate their phylogenetic utility at different taxonomic levels, we applied multivariate statistical methods on RE archives of *Rosaceae* with an emphasis on the genera *Fragaria* and *Rosa*, both being frequently affected by hybridization.

Our methodological approach was based on a modification of the phylogenetic applications of the RE pipeline (Novák et al., 2010) developed by Dodsworth et al. (2015, 2017) and Vitales et al. (2020). In these studies, the genomic abundance of a repeat was obtained by adjusting the read input to equal genome proportion for the respective species used in the comparative analysis. Additionally, Vitales et al. (2020) described dissimilarities between species by computing neighbor-joining trees based on the weighted amount of edges within a cluster (observed/expected number of edges). In contrast, we subsampled read numbers for each species in inverse proportionality to its genome size. Thus, in small genomes, we used a higher number of input reads than in large genomes (Figures 2A,B and Supplementary Table 1). In addition, we inverted and summarized all ratios of observed/expected number of edges across all clusters to consider the initial complete network in RE. This large network is an intermediate step during graph-based clustering and not in the final RE output (Yu et al., 2009; Novák et al., 2010, 2020). By applying this procedure, we detected strong phylogenetic signals within the *Rosaceae* data set and in *Fragaria* but less pronounced in *Rosa*.

In general, two factors (a biological and technical aspect) and their interplay could explain why the inversed read input is working. Technically, using direct proportional read input for calculating observed/expected edges matrices, the number of edges seems to be biased toward a higher self-interconnection in species with a higher number of reads. One reason might be that, in the all-to-all BLAST comparisons, similarity hits appeared more frequently between reads of the same species. Although the observed/expected number of edges ratios normalizes the similarity counts in RE, it still depends on read input, because the expected number of edges is directly dependent on the number of reads per cluster. The RE pipeline first generates clusters independently and only assigns species pairs later on. Therefore, the number of reads of a certain species in a cluster is dependent on the comparative read input amount. Another aspect could be related to the building algorithm for the main graph, generated by the iGraph package implemented in RE (Yu et al., 2009; Novák et al., 2010, 2020). Before clusters are separated by the Louvain method (Blondel et al., 2008), an initial complete network is computed based on the BLAST comparison with nodes and edges representing reads and weighted similarities, respectively. Inter-species connections are expected to have lower weight because of fewer similarities and are probably underrepresented if enough higher intra-species weights are available. Since we used the sum of inverted ratios of the observed/expected number of edges, our results more strongly reflected the large main network rather than decisions of modularity optimization by the Louvain method, which leads to specific clusters of repeat types. The RE algorithm did not differentiate between species (or individuals) at this point. It just displays shared repeat types. To overcome the biased “self-similarity,” a reduction of reads for larger genomes by indirect read input may be useful. This would increase the probability of inter-species connections, which is necessary to count edges reflecting similarities rather than nodes representing reads per repeat type abundance.

### Phylogenetic Patterns Within *Rosaceae*

Although our taxon sampling across *Rosaceae* was rather uneven and, by far, not comprehensive due to the limited availability of repeatome data, phylogenetic relationships were largely congruent to phylogenies calculated from plastomes (Zhang et al., 2017) and nuclear low copy genes (Xiang et al., 2016). Genera represented by several species always appeared as tight clusters and monophyletic groups (Figures 3, 4). The two larger subfamilies *Amgydaloideae* and *Rosoideae* were clearly separated from each other, and branching patterns within the subfamilies (tribes) corresponded with published phylogenies (Xiang et al., 2016; Zhang et al., 2017). However, within the *Amgydaloideae*, *Physocarpus opulifolius* (*Neilleae*) had an unsupported position in the analyses based on inverted and summarized observed/expected matrices (Figure 4) but formed the basal split in the analyses based on the actual edges distance matrix (Supplementary Figure 1), which is in accordance with previous phylogenies (Potter et al., 2007; Xiang et al., 2016; Zhang et al., 2017). Besides from nodes-defining subfamilies and genera, bootstrap support was rather low for some branches (e.g., *Geum*, *Physocarpus*, Figure 4 and Supplementary Figure 1). This result is concordant with phylogenies based on various plastid and nuclear markers obtained from Sanger Sequencing, which yielded also poorly supported nodes for the position of *Geum* and *Physocarpus* (Potter et al., 2007). In contrast, large phylogenies of *Rosaceae* obtained by High Throughput Sequencing yielded maximum support for nearly all nodes ([Bibr B96][Bibr B99]). However, high bootstrap values are likely to be observed in large data sets even when topologies were wrong or contradictory (Huang et al., 2021). Skipping numerous species- or lineage-specific clusters, because a pairwise matrix is not applicable or the matrix contains NAs, respectively, could be interpreted as information loss. However, it was our intention to analyze the similarity/dissimilarity of common and, probably, ancient ancestral repeats shared by many species. If we aimed only for species- and lineage-specific repeats, the direct proportional RE comparative analysis and the number of reads per cluster would be sufficient (repeat abundance can be found in the COMPARATIVE_ANALYSIS_COUNTS.csv file in the RE output archive, or read counts tables can be extracted with the bash script in Supplementary File 1). In general, we advocate that future studies should aim for a more balanced taxon sampling within *Rosaceae*, for instance, in the taxonomically challenging polyploid *Maleae* (Lo and Donoghue, 2012; Sun et al., 2018) to investigate whether similarity in repeatomes might provide useful insights for their phylogeny.

### Relationships Within *Fragaria*

In accordance with previous studies (Rousseau-Gueutin et al., 2009; Njuguna et al., 2013; Kamneva et al., 2017), all our analyses here revealed that *F. viridis* is clearly separated from the remaining species belonging to the Vesca clade of *Fragaria*. Samples of the naturally occuring hybrid *F.*×*bifera* (*F. vesca* × *F. viridis*) were in an intermediate position between *F. viridis* and species of the Vesca clade, whereas the triploid accession was closer to *F. viridis* in the analyses based on the complete repeatome and 45S rDNA (Figures 5A,B,C,D) but closer to *F. vesca* in the PCoA based on 5S rDNA (Figures 5E,F). Liu and Davis (2011) observed three loci of 5S rDNA and nine loci of 45S rDNA in triploid accessions of *F.*×*bifera*, implying that one locus of 5S and three loci of 45S rDNA exist per haploid genome. Taking into account that the here investigated 3x plant of *F.*×*bifera* contained the plastid DNA of *F. vesca* (Tushabe, 2019), this accession probably arose by an unreduced (2x) pollen grain of *F. viridis*.

In contrast, samples of the hexaploid *F. moschata* were not in proximity to *F. viridis* but clustered rather between *F. vesca* and the Asian diploid species *F. mandshurica*, *F. bucharica*, and the tetraploid *F. orientalis* (Figure 5). This contradicts the hypothesis that *F. moschata* is a polyploid derivative of *F.*×*bifera* (Staudt, 1959) and rather implies a hybridogenic origin within the Vesca clade. While *F. vesca* is being accepted as a parental species of *F. moschata*, the second parent has been controversially discussed. Some nuclear (*GBBS-2*: Rousseau-Gueutin et al., 2009; *adh*: DiMeglio et al., 2014) and plastid markers (Lin and Davis, 2000) proposed *F. viridis* as a potential parent, whereas other data (*DHAR*: Rousseau-Gueutin et al., 2009; target capture of nucelar low-copy genes: Kamneva et al., 2017) suggested *F. mandshurica* as a progenitor. Yang and Davis (2017) suggested that more than one diploid species may have been involved in the origin of the hexaploid *F. moschata*.

### Relationships Between *Rosa* Species

Previous studies indicated variable power of repeatomes in phylogenetic reconstructions. For example, it was highly efficient in *Nicotiana*, *Fritillaria*, *Fabaceae* (Dodsworth et al., 2015; Vitales et al., 2020), and in *Fragaria* (this study), while it did not lead to congruent phylogenies in other taxa (Vitales et al., 2020). Neither entire repeatome data nor 45S or 5S rDNA clusters reflected phylogenetic relationships within the genus *Rosa*. Plastid phylogenies supported the split of *Rosa* into two major clades: the *Rosa* and allies clade and the *Synstylae* and allies clade (Fougère-Danezan et al., 2015; Debray et al., 2019, 2021) with *R. persica* (subg. *Hulthemia*) as the most basal taxon, which was only separated from the remaining species in the PCoA and the neighbor net based on all clusters (Figures 7A,B). Our recent studies have revealed that the repeatome of roses, and, therein, especially satellite repeats, was little polymorphic between species. For example, the CANR4 satellite repeat appeared frequently at several loci across the entire genus but was absent in related genera (Lunerová et al., 2020). Diploid species contained less but more polymorphic CANR4 loci compared to the numerous polyploids in the genus (Lunerová et al., 2020). Interestingly, members of *Rosa* and *Asclepias* are perennial shrubs with complex evolutionary histories and a significant degree of intragenomic heterozygosity (Weitemier et al., 2015; Raymond et al., 2018). In both genera, repeatome-based phylogenies seem to be inconclusive or even providing erratic results (this work and Vitales et al., 2020). Perhaps, these factors, together with frequent polyploidization and hybridization events, may blur the phylogenetic signal of repeatomes. Rapid genome evolution after these events has also been reported (Parisod and Senerchia, 2012; Belyayev, 2014; Vicient and Casacuberta, 2017). Furthermore, the homoplasious nature of some repeat types and horizontal TE transfer could also obscure phylogenetic signals (Blumenstiel, 2019; Martín-Peciña et al., 2019). Although roses usually contain only one 45S rDNA locus per genome (Ma et al., 1997; Lim et al., 2005; Herklotz et al., 2018), its phylogenetic signal, mainly retrieved from ITS sequences, is rather limited due its high-sequence homogeneity across the genus (Matsumoto et al., 2000; Wu et al., 2001; Wissemann and Ritz, 2005). However, SNP-based analyses of ITS helped to investigate the origin of hybridogenic taxa (Ritz et al., 2005; Herklotz et al., 2018; see below). The evolution of the 5S rDNA in roses turned out to be complex because two early diverged variants coexist in various amounts across the genus (Vozárová et al., 2021). The pattern retrieved from the PCoA (Figure 7C) clearly reflects the proportion of A and B variants of 5S rDNA (Vozárová et al., 2021), namely that dogroses contain higher proportions of the A variant, which is typical for the *Rosa* and allies clade compared to the B variant, which is overrepresented in the *Synstylae* and allies clade. However, our comparative repeatome analysis of the 5S rDNA cluster in roses revealed more variants represented by at least four loops in the graphical display of the cluster (Figure 6B) and thus mixed signals of artificial recombination or several variants. Tandemly arranged satDNA sequences like the rose CANR4 and rDNA are often species- or genus-specific and are thought to be the most dynamic fraction, representing short-term evolutionary transition (Charlesworth et al., 1994; Raskina et al., 2008).

### Relationships With *Rosa* Subsect. *Caninae*

According to the results across the genus *Rosa*, neither entire repeatome data nor rDNA clusters give clear-cut insights into the relationships between species of sect. *Caninae* (Figure 7). The entire sect.*Caninae* originated by hybridization (Wissemann, 2000; Ritz et al., 2005); however, plastid phylogenies (Wissemann and Ritz, 2005; Bruneau et al., 2007; Fougère-Danezan et al., 2015) and experiments using fluorescent *in situ* hybridization of rDNA and the CANR4 satellite (Herklotz et al., 2018; Lunerová et al., 2020) revealed that subsect. *Caninae* and subsect. *Rubigineae* had independent origins *via* reciprocal hybridization events. Thus, the *Caninae* precursor genome forms bivalents in subsect. *Caninae* and univalents in subsect. *Rubigineae*, and, *vice versa*, the *Rubigineae* precursor genome forms bivalents in subsect. *Rubigineae* and univalents in subsect. *Caninae*. However, information from the probably different proportions of the different precursor genomes in the repeatome was not sufficient to differentiate between subsections. Moreover, subsections hybridize naturally: *R. dumalis* = subsect. *Caninae* × subsect. *Rubigineae* (Herklotz and Ritz, 2014) and *R. micrantha* (subsect. *Rubigineae* × subsect. *Caninae*; Ritz and Wissemann, 2011; Herklotz and Ritz, 2017), and these hybrids are expected to be strongly maternally biased due to the Canina meiosis (4/5 of the genome is inherited by the egg cell; Täckholm, 1922). Neither these natural occurring hybrids nor the respective synthetic hybrids clustered accordingly based on complete repeatome analysis (Figures 8A,B). However, 45S rDNA analysis retrieved the expected pattern because these samples had either an intermediate or matroclinal position in the PCoAs (Figure 8C). Samples of *R. rubiginosa* from different populations tended to cluster close to each other in both 45S and 5S graphs (Figures 8C,D,E,F); however, individuals of *R. canina* were scattered across the PCoAplots (Figures 8C,E). It has been repeatedly shown that species of subsect. *Caninae*, e.g., *R. canina*, represent genetically and morphologically more diverse species complexes compared to subsect. *Rubigineae*, here mainly represented by *R. rubiginosa* (Nybom et al., 1997; Werlemark et al., 2000; Jürgens et al., 2011; Herklotz and Ritz, 2017; Herklotz et al., 2017).

Remarkably, the taxonomic signal was strongly overlayed by a geographic signal in the rDNA data, especially in the 45S rDNA data. Irrespective of taxonomic affiliation, samples from Lower Saxony and from Eastern Saxony (E) were closely clustered (Figures 8C,D). Thus, ongoing genetic exchange, including backcrossing, in mixed dogrose populations might continuously blur species boundaries, a phenomenon also detected by Amplified Fragment Length Polymorphism in mixed populations of dogroses from Belgium (De Cock et al., 2008). This is in accordance with the finding that natural dogrose hybrids originate rather frequently and independently (Herklotz and Ritz, 2017).

### Advantages and Limitations of the “Repeatomic Fingerprint” Method

Previous studies demonstrated the usefulness of repeatomes for studies of phylogenetic inference (Dodsworth et al., 2015; Vitales et al., 2020). We have extended and improved this methodical approach in several aspects: (i) The algorithm uses an amount of data inversely proportional to sample genome size. We found that this operation is particularly useful when species differing in genome size while having overall genome similarity are compared. (ii) Our script is able to extract comparative matrices from the RepeatExplorer2 archives and to transform the data suitable for multivariate statistics. Such an approach might be convenient when a large number of species/genomes are analyzed in batch. (iii) Since we display the sum of all dissimilarities of common repeats, our method reflects a more global genomic relationship between taxa. (iv) Separate analyses of specific repeats like rDNA are made possible because our script uses text search in the RE archive, and, thus, subsampling could potentially be extended to clusters with hits to any other repeats (e.g., all Ty3/Gypsy annotations).

We admit the method has certain limitations, namely, that it, currently, cannot use clusters with missing nodes for one or more samples (annotated as “NA” in obs-matrices, species or lineage- specific absents). This presents a potential problem at higher taxonomic levels where differences between the genomes are high, and, thus, the number of useable clusters will be low. Further-on avoiding these clusters might reduce the phylogenetic information embedded in the clustering analysis. One future direction could be to replace NAs with artificial values, such as a one edge equivalent for the observed/expected ratios or a mean of the remaining values of the obs-matrix and to see how this impacts the topologies of graphs. We assume that species or lineage-specific clusters carry also a strong phylogenetic signal. Thus, we initially tested an abundance-based approach by concatenating the read counts of each cluster into a character matrix. However, subsequent multivariate analyses were blurred by signal noise from highly abundant repeats. Thus, future analyses may aim to filter for informative clusters, and, therefore, the reduction to specific repeat types or classes might be an option. The supplied bash script includes also the extraction of the read count tables per cluster (Line 76, Supplementary File 1 or in RE archive file “COMPARATIVE_ANALYSIS_COUNTS.csv”), but a direct proportional read input is important for abundance-based analyses and would change the whole statistical approach, which is beyond the scope of the paper.

Another challenge is low and approximately equal percentage of variances in all dimensions of the PCoAs in some analyses (e.g., in dogroses, Figure 7). However, even in these cases, the neighbor nets displayed exactly the same pattern compared to PCoA on the first two dimensions. Both statistical analyses differed in the treatment of obs-matrices. For neighbor nets, we used the obs-matrices as tables of variables, like Dodsworth et al. (2015) did this for read counts, and then, we calculated Gower distances based on these tables. In contrast, for PCoAs, obs-matrices were directly treated as distance according to Vitales et al. (2020). We think that range normalization implemented in the Gower distance has a major effect on the signal enhancement of the obs-matrices. Furthermore, differences between species were given as absolute values, avoiding negative branch lengths in neighbor nets. However, one can argue that the Gower distance calculation is a circular argument, as it uses all values as independent variables. Moreover, neighbor nets display all information from the data and not only the variance of the first two axes like in a PCoA. On the other hand, treating obs-matrices directly as distance has also pitfalls because it could happen that within-species distance is higher than between species in cases of species with very high-repeatome diversity. Since this could not be completely solved by our analyses, we would like to encourage further discussions on these statistical issues. In addition, future studies should also focus on the robustness of the method regarding taxon sampling. In general, phylogeneticists agree that denser taxon sampling will improve phylogenetic accuracy. However, this depends on the marker chosen and may not hold completely in fast-evolving markers with lineage-specific evolutionary rates (Nabhan and Sarkar, 2012). Therefore, the impact of taxon sampling might be shown by subdividing a large and comprehensive data set into various smaller ones. Using an amount of read data (inversely) proportional to sample genome size implements the assumption; that differences in genome size are independent from the effect of polyploidization (e.g., rearrangement, repeat expansion, and TE activity) and are linearly connected to repeat content, which is only approximately true (Choi et al., 2020). The genome size and its correct determination, ploidy level, and mode of polyploidization, together with the organization of repeats in the genomes, may play a role and should be also evaluated in further studies.

## Conclusion/Summary

During this study, we refined a method to track phylogenetic signals from repeatome data. The multivariate statistical approaches based on summed dissimilarities showed strong signals among larger taxonomic entities within the family *Rosaceae*. In *Fragaria*, a genus with restricted hybridization, we detected clear patterns of relatedness, including the correct position of hybrids. However, patterns were less pronounced in the more complicated genus *Rosa*, which is influenced by both recent and ancient hybridization. In contrast, both rDNA markers (5S or 45S) appeared to be informative in resolving species relationships in this group. It is, therefore, useful to analyze non-coding repeatomes and rDNA repeats from same source data sets separately. The described Principal Coordinate Analysis of repeatomes may provide a convenient approach to infer phylogenetic relationships, supplementing conventional methods, particularly in systems with complicated evolutionary histories. In the future, as high throughput sequencing is becoming more available to ancient DNA, our method could serve as an opportunity to analyze highly fragmented DNA by genome skimming from herbarium material.

## Data Availability Statement

The datasets presented in this study can be found in online repositories. The names of the repository/repositories and accession number(s) can be found in the article/Supplementary Material.

## Author Contributions

VH, AK, and CR conceived and designed the study and wrote the manuscript. VH, VW, JL, RV, SB, KO, and MG performed the experiments and collected material. VH analyzed the data.

## Conflict of Interest

KO is employed by Hansabred GmbH & Co. KG, Dresden, Germany. The remaining authors declare that the research was conducted in the absence of any commercial or financial relationships that could be construed as a potential conflict of interest.

## Publisher’s Note

All claims expressed in this article are solely those of the authors and do not necessarily represent those of their affiliated organizations, or those of the publisher, the editors and the reviewers. Any product that may be evaluated in this article, or claim that may be made by its manufacturer, is not guaranteed or endorsed by the publisher.

## Footnotes

1
https://www.geneious.com

2
https://galaxy-elixir.cerit-sc.cz/

## Supplementary Material

Supplementary Figure 1 Principal Coordinate Analysis (PCoA) of *Rosaceae* species based on the inverted and summarized edges matrices from comparative RepeatExplorer2 output.

Supplementary Figure 2 Neighbor-joining tree of *Rosaceae* species based on Gower distance calculated from the sum of 322 edges distances obtained from the comparative RepeatExplorer2 output. The tree was rooted with *Dryas drummondii*, and Bootstrap support >50% is given above branches.

Supplementary Table 1 Analyzed species, including taxonomic information of four different data sets with respective indirect proportional RepeatExplorer2 read inputs according to genome size. The genome size as 2C value, its source, and the Short Read Archive (SRA) accession numbers are given.

Supplementary File 1 Bash script to be executed from the parent directory of the decompressed RepeatExplorer2 archive to produce txt-files containing numbers of edges between species matrices (edges), observed/expected number of edges between species matrices (obs), and species read counts (reads).

Supplementary File 2 R script to invert and summarize observed/expected number of edges between species matrices (obs) and numbers of edges between species matrices (edges).
