# Supplementary material for: Power and Weakness of Repetition – Evaluating the Phylogenetic Signal From Repeatomes in the Family Rosaceae With Two Case Studies From Genera Prone to Polyploidy and Hybridization (Rosa and Fragaria)
Source: Front Plant Sci. 2021 Dec 7;12:738119. doi: 10.3389/fpls.2021.738119 (PMC8688825; doi:10.3389/fpls.2021.738119)
Supplement: Supplementary Figure 1 — Principal Coordinate Analysis (PCoA) of Rosaceae species based on the inverted and summarized edges matrices from comparative RepeatExplorer2 output. [file Data_Sheet_1.zip › Supplementary Figure 2.pdf]

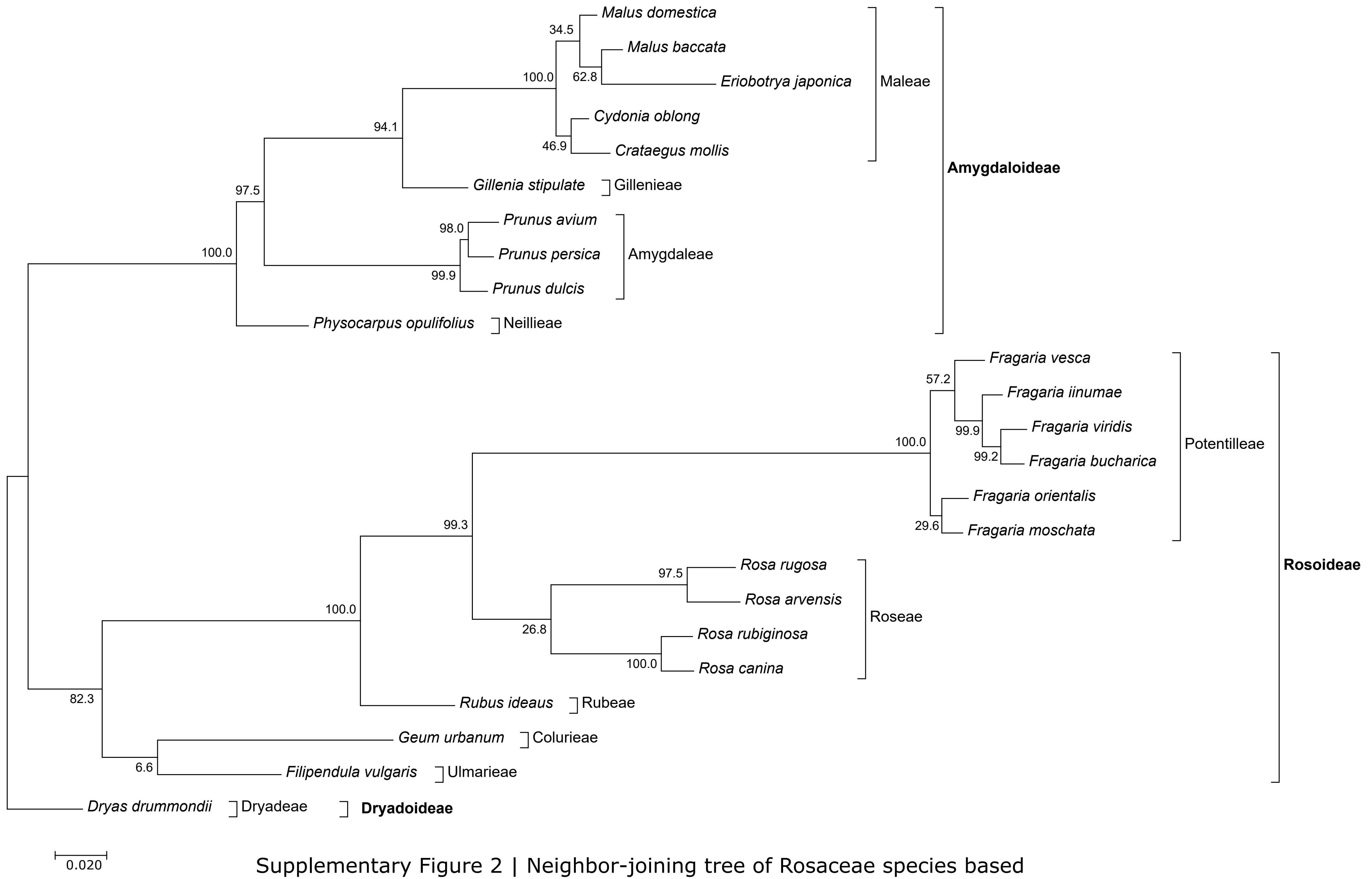

Supplementary Figure 2 | Neighbor-joining tree of Rosaceae species based on Gower distance calculated from the sum of 322 edges distances obtained from the comparative RepeatExplorer2 output. The tree was rooted with *Dryas drummondii*, and Bootstrap support >50% is given above branches.
